# More Health Co-Ordination Needed

**Published:** 1920-03-20

**Authors:** 


THE PUBLIC WELL-BEING—(*continued*).

### More Health Co-ordination Needed.

None of the great cities appears to have followed Leeds, or to have taken action such as Leeds has done regarding the lamentable disclosures in the report on recruits examined for the Army. This is disappointing, for, though Leeds was shown to be one of the dark spots, it was not shown to be the worst, and the public spirit and enterprise of a great paper like the *Yorkshire Post* in sifting the report might well have been expected also in the *Birmingham Post*, the *Manchester Guardian*, the *Sheffield Telegraph*, the *Liverpool Post*, etc., and the leading Welsh and Scottish papers. It is true the Press of the whole country published long extracts, but none seems to have explored the whole local situation like the Leeds papers. They have examined every phase of local welfare administration; they have had several leading articles; they have interviewed all who could throw any light on the matter, and they have informed public opinion upon the present shortcomings, as well as upon the immediate and future needs.

A sane and educated public opinion is the only solid foundation on which to build. One suggestion which has been surveyed is the co-ordination from a common centre under the direct guidance of the local authority of the twenty odd voluntary organisations in Leeds concerned with child welfare, so that overlapping may be avoided. Such amalgamation was recommended in the last annual report of the Chief Medical Officer of Health of the Board of Education, and it has the endorsement of the Leeds school medical officer. In Leeds the school medical officer's department is separate and independent from that of the medical officer of health. It is the same in Sheffield and Manchester, but in London, Birmingham, and Liverpool the medical officer is responsible for the medical inspection of children. The medical officer of health should be the head of the health administration in every city, and be responsible to the local authority. It would not be necessary to deprive the voluntary organisations of their special character if they became co-ordinated under the guidance of an experienced expert with all official information which could be placed at their disposal, enabling them to direct their energies towards definite and particular ends.

The subject of impressing public opinion needs repeated emphasis, not from the point of view only of those who should be called in to assist in pushing forward general welfare, but from the point of view also of those who are uninterested, ignorant, and careless. There is much keener corporate interest in health than there has ever been, but the power of the slack individual to inflict harm upon others is so wide that observance of the elementary things must be drummed into his thick head, and he should be made to understand by punishment and all possible social pressure that he cannot be allowed to continue a nuisance and a danger to himself and his fellows. He ignores the need of exercise and physical culture. He dulls his whole system by fear of the cheapest of all tonics—fresh air. He over-eats, over-drinks, over-sleeps, and over-tires himself. Nature exacts retribution for every physical abuse. It was pointed out by an authority the other day that it was astonishing to find how many people suffering from tuberculosis or other diseases had not the faintest knowledge of the symptoms, and who apparently never bothered to have themselves medically overhauled. There is always the temptation of the moralist to say—when it is safe to do so—that the neglectful deserve to suffer the consequences; but that is not a standpoint which commands much support, and it cannot command support at all where it involves the health of the community. Take only the case of tuberculosis. How much disease has been spread by lack of simple ventilation, and, worse still, by the disgusting habit of expectoration? The county tuberculosis officer for Cornwall said the other day that "the great predominating channel of infection was the sputum of well-developed cases; stop the source of infection and the disease was wiped out as an appreciable factor for death and suffering from the community." The habit should be made punishable everywhere, and punishment should be insisted on. Possibly health education in the schools may have its effect on coming generations, but a thing which has not yet been tried and which might be useful is a national advertising campaign, with the broad elementary facts and advice displayed in simple, popular language that will attract the eye and reach the intelligence of the feeblest and laziest minded. It was done to raise war loans and to get recruits; it is done in the "Safety first" campaign in the streets; why can't it be tried for health?

